# A heart failure phenotype stratified model for predicting 1-year mortality in patients admitted with acute heart failure: results from an individual participant data meta-analysis of four prospective European cohorts

**DOI:** 10.1186/s12916-020-01894-2

**Published:** 2021-01-27

**Authors:** Yuntao Chen, Adriaan A. Voors, Tiny Jaarsma, Chim C. Lang, Iziah E. Sama, K. Martijn Akkerhuis, Eric Boersma, Hans L. Hillege, Douwe Postmus

**Affiliations:** 1 Department of Epidemiology, University of Groningen, University Medical Center Groningen, Hanzeplein 1, P.O. Box 30.001, 9700 RB Groningen, the Netherlands; 2 Department of Cardiology, University of Groningen, University Medical Center Groningen, Groningen, the Netherlands; 3 Department of Social and Welfare Studies, Faculty of Health Sciences, Linköping University, Linköping, Sweden; 4 Division of Molecular and Clinical Medicine, University of Dundee, Ninewells Hospital and Medical School, Dundee, UK; 5 Department of Cardiology, Thoraxcenter, Erasmus Medical Centre, Rotterdam, the Netherlands

**Keywords:** Acute heart failure, Mortality, IPD meta-analysis, Prognostic model

## Abstract

**Background:**

Prognostic models developed in general cohorts with a mixture of heart failure (HF) phenotypes, though more widely applicable, are also likely to yield larger prediction errors in settings where the HF phenotypes have substantially different baseline mortality rates or different predictor-outcome associations. This study sought to use individual participant data meta-analysis to develop an HF phenotype stratified model for predicting 1-year mortality in patients admitted with acute HF.

**Methods:**

Four prospective European cohorts were used to develop an HF phenotype stratified model. Cox model with two rounds of backward elimination was used to derive the prognostic index. Weibull model was used to obtain the baseline hazard functions. The internal-external cross-validation (IECV) approach was used to evaluate the generalizability of the developed model in terms of discrimination and calibration.

**Results:**

3577 acute HF patients were included, of which 2368 were classified as having HF with reduced ejection fraction (EF) (HFrEF; EF < 40%), 588 as having HF with midrange EF (HFmrEF; EF 40–49%), and 621 as having HF with preserved EF (HFpEF; EF ≥ 50%). A total of 11 readily available variables built up the prognostic index. For four of these predictor variables, namely systolic blood pressure, serum creatinine, myocardial infarction, and diabetes, the effect differed across the three HF phenotypes. With a weighted IECV-adjusted AUC of 0.79 (0.74–0.83) for HFrEF, 0.74 (0.70–0.79) for HFmrEF, and 0.74 (0.71–0.77) for HFpEF, the model showed excellent discrimination. Moreover, there was a good agreement between the average observed and predicted 1-year mortality risks, especially after recalibration of the baseline mortality risks.

**Conclusions:**

Our HF phenotype stratified model showed excellent generalizability across four European cohorts and may provide a useful tool in HF phenotype-specific clinical decision-making.

## Background

Heart failure (HF) is a rapidly growing public health concern with high prevalence, poor prognosis, and high cost. It is estimated that 0.4–2.2% of the population in industrialized countries suffer from HF, with 500k–600k incident cases diagnosed each year [1]. Data from the 2016/2017 UK National Heart Failure Audit [2] showed that mortality remains high with in-hospital mortality and 1 year post-discharge mortality rates of 9.4% and 23.3%, respectively. The total medical expenditure on HF is predicted to rise from US$20.9 billion to $53.1 billion, of which 80% are attributed to increased hospitalization [3]. All of the aforementioned statistics will even deteriorate with the global aging. Accurately predicting prognosis for HF can help in tailoring treatments to subgroups of patients, as was recently shown for the selective adenosine A1 receptor antagonist rolofylline [4] as well as for the disease management programs evaluated in the COACH study [5].

Many clinical prediction models have been developed with the goal of helping physicians stratify patients with HF [6]. Some of these models were developed in patient populations with a particular HF phenotype, such as the Seattle Heart Failure Model (SHFM) [7] that was developed in the setting of HF with reduced ejection fraction (HFrEF), while others were developed in more general cohorts with a mixture of HF phenotypes, such as the MAGGIC risk score [8]. While such latter heterogeneous population models are more widely applicable, they are also likely to yield larger prediction errors for two reasons. One is the potential different baseline mortality rates of three HF subtypes, as indicated by several large studies [9, 10] that mortality of HF with preserved ejection fraction (HFpEF) is lower than that in HFrEF, even after adjusting for age, sex, and clinical covariates. However, a recent meta-analysis [11] showed no significant difference in mortality rates between HFrEF and HFpEF. The other one is the potential different predictor-outcome associations across HF subtypes. Among those, age, systolic blood pressure (SBP), and diabetes were verified by large cohort studies [8, 12] to have different associations with mortality in patients with HFrEF and HFpEF. Reducing uncertainty in risk prediction model by addressing the aforementioned two factors is essential to improve the prediction accuracy, which could in turn lead to improvements in advanced care planning, treatment adherence, and integration with wider healthcare teams such as palliative care. The purpose of this study was to use individual participant data (IPD) meta-analysis to develop an HF phenotype stratified model for predicting 1-year mortality in patients admitted with acute HF.

## Methods

### Study cohorts

Four cohorts were included in the IPD meta-analysis: BIOSTAT-index, BIOSTAT-validation, THRIUMPH, and COACH (Table 1). Detailed inclusion and exclusion criteria of the four cohorts are provided in Table S1 in Additional file 1. In short, BIOSTAT-CHF [13] was a large European project aimed to characterize biological pathways related to response or non-response to the recommended therapy for HF. To characterize these pathways, two independent HF cohorts were assembled: an index cohort (BIOSTAT-index) consisting of 2516 patients from 69 centers in 11 European countries and a validation cohort (BIOSTAT-validation) consisting of 1738 patients from 6 centers in Scotland, UK. TRIUMPH [14] was a translational bench-to-bedside study program encompassing the entire spectrum of biomarker discovery to clinical validation. The clinical validation study was an observational prospective study that enrolled 475 patients admitted with acute HF from 14 centers in the Netherlands. This study was designed to establish the clinical value of biomarkers successfully passing the bio-informatics and early-validation stages of TRIUMPH as well as to further evaluate more established biomarkers of HF. COACH [15] was a multicenter randomized controlled trial (RCT) that enrolled 1023 patients admitted with acute HF. This study was designed to evaluate the long-term effects of moderate or intensive disease management on outcome in patients with HF. All patients provided written informed consent. This study was conducted in compliance with the Declaration of Helsinki and was approved by all relevant local ethics committees.

**Table 1:** Detailed information for four included cohorts

ID	Study	***N***	Period	Study type	Site	Median follow-up (months)	Primary outcomes
1	BIOSTAT-index	2516	2010–2012	Cohort	69 centers in 11 European countries	21	Time to composite death or unscheduled hospitalizations for HF
2	BIOSTAT-validation	1738	2010–2014	Cohort	6 centers in Scotland	21	Time to composite death or unscheduled hospitalizations for HF
3	TRIUMPH	475	2009–2012	Cohort	14 centers in the Netherlands	10.8	All-cause mortality and readmission for HF
4	COACH	1023	2002–2007	RCT	17 centers in the Netherlands	18.4	Time to death or rehospitalization because of HF

Patients who were enrolled from outpatient clinics (*N* = 1625), had missing outcome data (*N* = 29), or had missing ejection fraction values (*N* = 459) were successively excluded for the present analysis. This resulted in a total sample of 3577 patients, of which 2368 were HFrEF patients, 588 were HF with midrange ejection fraction (HFmrEF) patients, and 621 were HFpEF patients. The HF subtypes were defined according to the European Society of Cardiology guidelines: HFrEF as < 40%, HFmrEF as 40–49%, and HFpEF as ≥ 50% [16].

### Outcome and predictor variables

The outcome of interest was 1-year mortality, defined as the time from hospital admission to death from any cause within 1 year after hospital admission. The candidate predictor variables consisted of a set of demographic, clinical, and laboratory variables that were selected according to clinical knowledge, literature [6], and data availability. This included age, sex, myocardial infarction (MI), atrial fibrillation, COPD, peripheral arterial disease, stroke, diabetes, previous HF hospitalization, NYHA class, SBP, diastolic blood pressure, heart rate, BMI, hemoglobin, N-terminal pro-B-type natriuretic peptide (NT-proBNP), serum potassium, serum sodium, serum creatinine, blood urea nitrogen (BUN), coronary artery bypass grafting (CABG), and implantable cardioverter defibrillator (ICD) or pacemaker. Medication use were excluded from the candidate variables because they might be confounded by disease severity influencing tolerability of the use [17]. For the clinical and laboratory variables, the measurements closest to the day of hospital admission were taken. Since patients who died during the index admission were excluded from the COACH study [15], the survival times for patients in COACH were left-truncated at the time of hospital discharge.

### Model derivation

Our prognostic model consists of two parts: (i) a prognostic index (PI) that captures the effects of the predictor variables, and (ii) HF subtype (HFrEF, HFmrEF, and HFpEF) specific baseline hazard functions that determine the baseline mortality rates in these three subpopulations.

Following Royston et al. [18], the PI was estimated from a Cox model stratified by cohort and HF subtype. First, a full model with all the predictors and their interaction with HF subtype was built. Backward elimination was then applied to the interaction terms. Another round of backward elimination was subsequently applied to the main effect terms, with the main effects of variables with significant interaction terms retained in the model. The significance level to stay in the model was set to .05. The counting process method was used to account for the left-truncated time-to-event data in COACH [19]. Missing values for the predictor variables were handled using multiple imputation with Rubin’s rules applied to obtain pooled estimates and *P* values at each step of the two backward elimination procedures [20]. Fractional polynomials were used to check the linearity of continuous predictors and to find suitable transformations in case the linearity assumption did not hold [21].

The baseline hazard functions were obtained by fitting an HF subtype stratified Weibull model to the pooled data with the PI obtained from the Cox model included as an offset. The full parameterization of our HF subtype stratified prognostic model can be found in Additional file 2.

### Model validation

Model performance was assessed in terms of discrimination and calibration [22]. Discrimination was assessed using the area under the cumulative/dynamic time-dependent ROC (AUC) computed at the evaluation time of 1 year [23]. Calibration was assessed by calibration plots comparing predicted vs. observed 1-year mortality rates in total and in subgroups with different predicted risks.

To evaluate the generalizability of our prognostic model, both raw AUCs and internal-external cross-validated AUCs were computed. The internal-external cross-validation (IECV) approach was also used for generating the calibration plots. IECV is a sequential approach in which every study is excluded once to serve as an external validation cohort for a prognostic model developed in the remaining three cohorts [24]. In this way, it can be evaluated whether the derived model has good prognostic separation in independent cohorts and whether the baseline mortality is comparable across study populations.

### Comparison with other risk scores

To compare the predictive performance of our model with the predictive performance of three existing risk scores, namely the MAGGIC risk score [8], the GWTG-HF score [25], and the BCN Bio-HF Calculator [26], the AUCs of these three models were compared with the internal-external cross-validated AUCs of our model. Calculations were performed separately for each of the four study cohorts and stratified by HF phenotype.

## Results

### Patient population

Baseline characteristics stratified by study cohort are provided in Table 2. BIOSTAT-validation had the largest proportion of HFmrEF and HFpEF patients, while these two subpopulations were underrepresented in BIOSTAT-index. Compared to the other three cohorts, COACH had fewer NYHA class I/II patients and more NYHA class IV patients. TRIUMPH had the smallest proportion of patients with previous HF hospitalization compared to the other three cohorts. Concerning medical history, TRIUMPH had a larger proportion of patients with CABG or ICD/pacemaker, while COACH had a smaller proportion of patients with diabetes. Concerning medication use before admission, a larger proportion of patients in BIOSTAT-index used β-blockers or ACE/ARBs. A smaller proportion of patients in COACH used β-blockers or ACE/ARBs. Diuretics were used by almost all the patients in BIOSTAT-index and BIOSTAT-validation. The distributions of the other variables were comparable across the four cohorts.

**Table 2:** Basic characteristics

	BIOSTAT-index (***n*** = 1469)	BIOSTAT-validation (***n*** = 809)	TRIUMPH (***n*** = 372)	COACH (***n*** = 927)	Overall (***n*** = 3577)
**Characteristics**					
Female sex	407 (27.7%)	309 (38.2%)	135 (36.3%)	344 (37.1%)	1195 (33.4%)
Age, mean (SD), years	68.1 (12.4)	74.7 (10.8)	70.7 (12.3)	70.5 (11.4)	70.5 (12.0)
BMI, mean (SD)	27.8 (5.61)	28.6 (6.63)	28.3 (5.54)	26.2 (5.15)	27.6 (5.80)
Blood pressure, mean (SD), mmHg					
Systolic	124 (22.0)	122 (22.3)	131 (28.8)	118 (21.0)	122 (22.9)
Diastolic	73.9 (13.3)	66.5 (13.5)	76.2 (17.3)	68.5 (12.1)	71.1(14.0)
Heart rate, mean (SD)	82.5 (20.5)	77.0 (17.5)	88.1 (22.3)	74.4 (13.4)	79.7 (18.9)
Previous HF hospitalization	419 (28.5%)	234 (28.9%)	80 (21.5%)	293 (31.6%)	1026 (28.7%)
NYHA class					
I/II	424 (28.9%)	201 (24.8%)	67 (18.0%)	49 (5.3%)	741 (20.7%)
III	756 (51.5%)	407 (50.3%)	193 (51.9%)	477 (51.5%)	1833 (51.2%)
IV	249 (17.0%)	201 (24.8%)	93 (25.0%)	393 (42.4%)	936 (26.2%)
HF subtypes					
HFrEF	1159 (78.9%)	332 (41.0%)	254 (68.3%)	623 (67.2%)	2368 (66.2%)
HFmrEF	187 (12.7%)	201 (24.8%)	54 (14.5%)	146 (15.7%)	588 (16.4%)
HFpEF	123 (8.4%)	276 (34.1%)	64 (17.2%)	158 (17.0%)	621 (17.4%)
**Medical history**					
Myocardial infarction	513 (34.9%)	409 (50.6%)	141 (37.9%)	387 (41.7%)	1450 (40.5%)
CABG	244 (16.6%)	133 (16.4%)	103 (27.7%)	149 (16.1%)	629 (17.6%)
Atrial fibrillation	681 (46.4%)	372 (46.0%)	153 (41.1%)	410 (44.2%)	1616 (45.2%)
ICD/pacemaker	336 (22.9%)	83 (10.3%)	111 (29.8%)	79 (8.5%)	609 (17.0%)
COPD	264 (18.0%)	184 (22.7%)	68 (18.3%)	237 (25.6%)	753 (21.1%)
Peripheral arterial disease	173 (11.8%)	161 (19.9%)	81 (21.8%)	155 (16.7%)	570 (15.9%)
Stroke	136 (9.3%)	176 (21.8%)	67 (18.0%)	144 (15.5%)	523 (14.6%)
Diabetes	505 (34.4%)	281 (34.7%)	132 (35.5%)	254 (27.4%)	1172 (32.8%)
Medication*					
β-Blocker use	1164 (79.2%)	562 (69.5%)	231 (62.1%)	427 (46.1%)	2384 (66.6%)
ACE/ARBs use	1007 (68.6%)	504 (62.3%)	231 (62.1%)	463 (49.9%)	2205 (61.6%)
Diuretics use	1467 (99.9%)	800 (98.9%)	261 (70.2%)	692 (74.6%)	3220 (90.0%)
**Laboratory, mean (SD)**					
Hemoglobin, mmol/L	8.14 (1.20)	7.92 (1.30)	8.17 (1.30)	8.39 (1.22)	8.16 (1.25)
Hematocrit, %	39.8 (5.41)	39.9 (6.20)	40.0 (6.06)	41.0 (5.81)	40.1 (5.79)
Serum potassium, mmol/L	4.21 (0.58)	4.18 (0.50)	4.24 (0.64)	4.21 (0.61)	4.21 (0.58)
Serum sodium, mmol/L	139 (4.07)	138 (3.63)	139 (4.12)	138 (4.66)	139 (4.15)
Serum creatinine, μmol/L	115 (52.3)	111 (51.7)	126 (63.7)	123 (54.0)	117 (54.1)
BUN, mmol/L	16.4 (13.1)	10.6 (6.20)	12.0 (9.76)	10.7 (5.64)	13.0 (10.1)
NT-proBNP, ng/L	7670 (8830)	4990 (8080)	6910 (7650)	4960 (6980)	6080 (8120)
**Death**^†^					
HFrEF	195 (16.8%)	94 (28.3%)	54 (21.3%)	126 (20.2%)	469 (19.8%)
HFmrEF	40 (21.4%)	42 (20.9%)	11 (20.4%)	28 (19.2%)	121 (20.6%)
HFpEF	26 (21.1%)	69 (25.0%)	11 (17.2%)	22 (13.9%)	128 (20.6%)

*Medication use was assessed prior to hospital admission

^†^Number (%) of death within 1 year of follow-up by study cohort and HF phenotype

The extent of missing data for baseline characteristics is provided in Table S2 in Additional file 1. The proportion of missing data for most of the candidate predictors was very small (< 2%). BUN and NT-proBNP had a relatively larger proportion of missing data (6.7% and 33.2%, respectively).

Within 1 year of follow-up, the number of mortality events was 469 (19.8%) in patients with HFrEF, 121 (20.6%) in patients with HFmrEF, and 128 (20.6%) patients with HFpEF (Table 2).

### Clinical prediction model

The final model included 11 predictors: age, COPD, NYHA class, hemoglobin, serum sodium, BUN, NT-proBNP, SBP, serum creatinine, MI, and diabetes. Four of these predictors, namely SBP, serum creatinine, MI, and diabetes, interacted with HF subtype. SBP, BUN, serum creatinine, and NT-proBNP were transformed because of non-linear relationships with mortality. The relative effects of the predictors after transformation are presented in Table 3.

**Table 3:** Results from multivariable Cox regression stratified by study cohort and HF subtype

Variables	Transformation	Coef (SE)	HR (95% CI)	*P*_interaction_
Age, year		0.023 (0.004)	1.02 (1.02–1.03)	
COPD		0.298 (0.087)	1.35 (1.14–1.60)	
NYHA class III		0.360 (0.124)	1.43 (1.12–1.83)	
NYHA class IV		0.298 (0.133)	1.35 (1.04–1.75)	
Hemoglobin, mmol/L		− 0.164 (0.034)	0.85 (0.79–0.91)	
Sodium, mmol/L		− 0.032 (0.009)	0.97 (0.95–0.99)	
BUN, mmol/L	= log2(x*)	0.335 (0.065)	1.40 (1.23–1.59)	
NT-proBNP, ng/L	= log2(*x*)	0.294 (0.035)	1.34 (1.25–1.44)	
SBP (HFrEF), mmHg	= min(*x*,130)^†^	− 0.029 (0.003)	0.97 (0.96–0.98)	< .001
SBP (HFmrEF), mmHg	= min(*x*,130)	− 0.009 (0.008)	0.99 (0.98–1.01)	
SBP (HFpEF), mmHg	= min(*x*,130)	− 0.006 (0.007)	0.99 (0.98–1.01)	
Creatinine (HFrEF), μmol/L	= log2(*x*)	0.037 (0.100)	1.04 (0.85–1.26)	.010
Creatinine (HFmrEF), μmol/L	= log2(*x*)	− 0.367 (0.140)	0.69 (0.53–0.91)	
Creatinine (HFpEF), μmol/L	= log2(*x*)	− 0.209 (0.150)	0.81 (0.54–1.19)	
MI (HFrEF)		0.430 (0.097)	1.54 (1.27–1.86)	.001
MI (HFmrEF)		− 0.032 (0.188)	0.97 (0.67–1.40)	
MI (HFpEF)		− 0.216 (0.201)	0.79 (0.53–1.17)	
Diabetes (HFrEF)		0.265 (0.101)	1.30 (1.07–1.59)	.041
Diabetes (HFmrEF)		− 0.176 (0.202)	0.84 (0.56–1.25)	
Diabetes (HFpEF)		− 0.077 (0.190)	0.93 (0.64–1.34)	

*x stands for original value

^†^The SBP has a linear trend up to 130 mmHg, while above 130 mmHg the risk is constant. Therefore, we truncated SBP at 130 mmHg

**Fig. 1:**
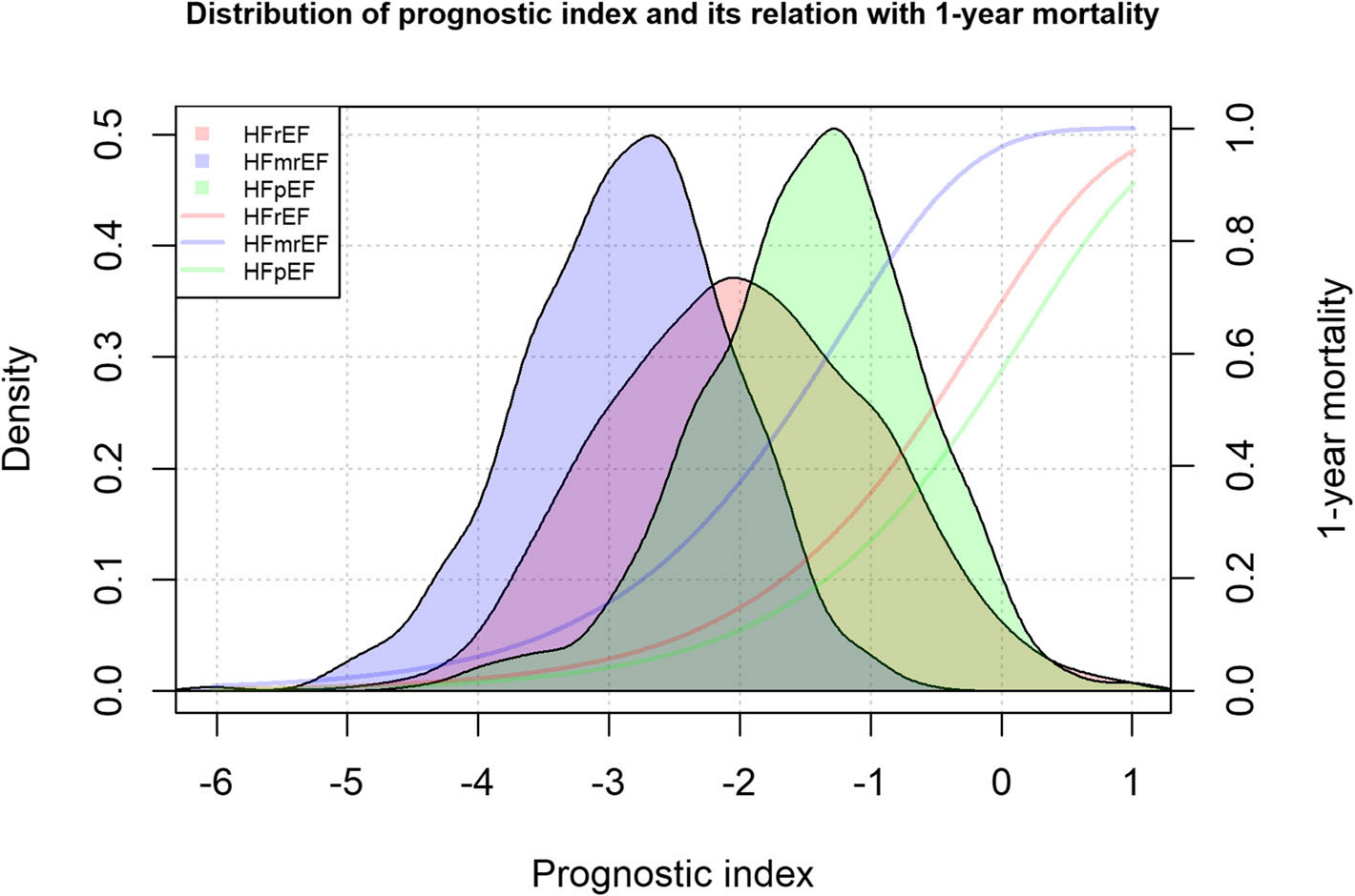
Distribution of prognostic index and its relation with 1-year mortality in HFrEF, HFmrEF, and HFpEF

The PI for a specific patient is calculated as the linear combination of the regression coefficients (Table 3) and the values of the corresponding (transformed) predictors for that patient. The distribution of the PI in the pooled dataset is presented in Fig. 1, which also shows the predicted 1-year mortality risk associated with the different values of the PI stratified by HF subtype. Specifically, the median and interquartile range of the PI was − 2.0 (− 2.7 to − 1.3) for HFrEF, − 2.8 (− 3.4 to − 2.3) for HFmrEF, and − 1.4 (− 2.0 to − 0.9) for HFpEF, which associated 1-year predicted mortality risks of 14.8% (7.6 to 28.3%), 18.5% (10.8 to 28.4%), and 18.5% (11.3 to 28.9%) for HFrEF, HFmrEF, and HFpEF, respectively. The mathematical formulas underlying the predicted 1-year mortality risk curves shown in Fig. 1 are provided in Additional file 3 together with an illustration of how these calculations can be conducted for an example patient.

### Model validation

The raw AUCs (95% CIs) for HFrEF, HFmrEF, and HFpEF were 0.78 (0.76–0.81), 0.75 (0.70–0.80), and 0.74 (0.70–0.79), respectively. The IECV approach yielded comparable estimates, with a weighted IECV-adjusted AUC (95% CI) of 0.79 (0.74–0.83) for HFrEF, 0.74 (0.70–0.79) for HFmrEF, and 0.74 (0.71–0.77) for HFpEF. Moreover, the relatively small differences between the estimated and predicted AUCs for the individual cohorts in the IECV showed that the discrimination reproduced well across four cohorts (Table 4).

**Table 4:** Evaluation of heterogeneity of AUC across four studies (internal-external cross-validation)

Study(*k*)	HFrEF		HFmrEF		HFpEF	
	AUC_(*k*)_*	AUC_*k*_^†^	AUC_(*k*)_	AUC_*k*_	AUC_(*k*)_	AUC_*k*_
BIOSTAT-index	0.80	0.76	0.73	0.77	0.76	0.70
BIOSTAT-validation	0.77	0.84	0.79	0.69	0.76	0.75
TRIUMPH	0.78	0.80	0.75	0.80	0.75	0.68
COACH	0.79	0.76	0.75	0.74	0.73	0.78
Total^‡^	0.79	0.79	0.76	0.74	0.75	0.74

*AUC estimated in other three cohorts after omitting study *k*

^†^AUC predicted in study *k*

^‡^Weighted mean across four cohorts. See Royston et al. [18]

**Fig. 2:**
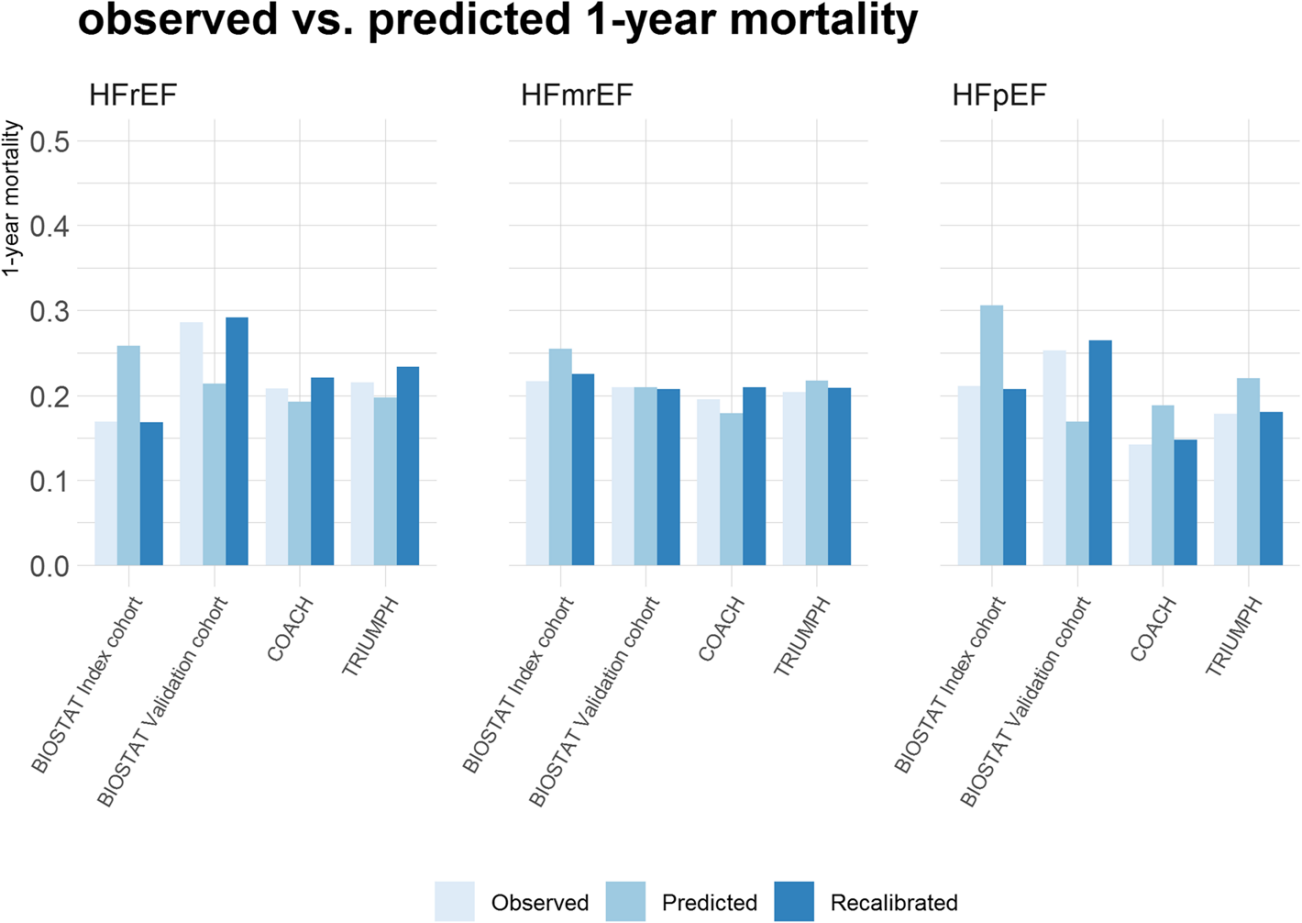
Internal-external cross-validation-based cohort-specific observed vs. predicted 1-year mortality in HFrEF, HFmrEF, and HFpEF. Recalibrated refers to the predicted 1-year mortality after recalibration of the baseline mortality risks

In the pooled dataset, the average observed vs. predicted 1-year mortality rates were 20.3% vs. 20.6% for HFrEF, 21.2% vs. 21.5% for HFmrEF, and 21.3% vs. 21.6% for HFpEF. The results of the IECV showed that the discrepancies between the observed vs. predicted 1-year mortality rates were larger for the four individual cohorts (Fig. 2), especially for BIOSTAT-index and BIOSTAT-validation. These discrepancies disappeared after recalibration of the baseline mortality risks in each of the omitted cohorts [27] (Fig. 2). Calibration plots comparing the average observed vs. predicted 1-year mortality in different risk groups (deciles of predicted 1-year mortality in HFrEF and quintiles of predicted 1-year mortality in HFmrEF and HFpEF) yielded similar findings (Additional file 1: Figures S1–S6).

### Comparison with other risk scores

In HFrEF, our model outperformed the three existing risk scores. In HFmrEF and HFpEF, the BCN Bio-HF score showed a similar performance to our model, while the predictive performance of the MAGGIC score and the GWTG-HF score was lower (Table 5).

**Table 5:** Comparison of internal-external cross-validated AUC of our model with AUCs of the MAGGIC, GWTG-HF, and BCN Bio-HF risk scores in each of the study cohorts stratified by HF subtype

Study	HFrEF				HFmrEF				HFpEF			
	MAGGIC	GWTG-HF	BCN Bio-HF	Our model	MAGGIC	GWTG-HF	BCN Bio-HF	Our model	MAGGIC	GWTG-HF	BCN Bio-HF	Our model
BIOSTAT-index	0.71	0.69	0.70	0.73	0.71	0.66	0.76	0.73	0.50	0.53	0.66	0.67
BIOSTAT-validation	0.78	0.77	0.78	0.83	0.69	0.66	0.75	0.67	0.66	0.65	0.72	0.70
TRIUMPH	0.73	0.71	0.72	0.78	0.64	0.57	0.84	0.79	0.63	0.66	0.69	0.65
COACH	0.66	0.70	0.69	0.76	0.64	0.62	0.67	0.70	0.72	0.68	0.75	0.72

## Discussion

Using data collected from 3577 patients across four European cohorts, we developed an HF phenotype stratified model for predicting 1-year mortality in patients hospitalized because of acute HF. All the 11 predictors in the model should be readily available in routine clinical practice worldwide. Four of the predictors, namely SBP, serum creatinine, MI, and diabetes, influenced mortality risk differently in the HF phenotypes. For the other 7 variables, the effect on mortality was the same across the three phenotypes. The results of the IECV showed excellent discrimination with a weighted IECV-adjusted AUC of 0.79 (0.74–0.83) for HFrEF, 0.74 (0.70–0.79) for HFmrEF, and 0.74 (0.71–0.77) for HFpEF. These results also showed a good agreement between the average observed and predicted 1-year mortality risks, especially after recalibration to the cohort-specific baseline risks.

The vast majority of the existing prediction models were derived using data from a single HF cohort and then either internally validated or externally validated using data from a second HF cohort. An alternative approach that makes better use of the available data is to perform IPD meta-analysis [24]. While the use of IPD meta-analysis can result in more generalizable prediction models [28], this approach has so far only been applied for the MAGGIC risk score [8], which was predominately developed in ambulatory HF patients. To our knowledge, our study was the first IPD meta-analysis to develop an HF phenotype stratified model in the setting of acute HF. By including patients from three prospective cohorts and one RCT across Europe, the patient population used to develop our model was relatively broad and heterogeneous, and closer to routine clinical practice, especially compared to previous models that were derived from a single HF cohort. Our model nevertheless still showed a very good discriminative ability, with IECV-adjusted AUC of 0.79 for HFrEF, 0.74 for HFmrEF, and 0.74 for HFpEF. The discriminative ability of our model is promising as compared to mean c-statistics of 0.71 across 117 models for predicting mortality in a meta-analysis [6].

Our model outperformed the MAGGIC risk score, especially in HFrEF, indicating that the MAGGIC risk score might be not applicable for patients with decompensated HF, but more suitable for patients with a stable state. It is not unexpected that our model was also better than the GWTG-HF risk score since the latter was initially developed to predict in-hospital mortality. The BCN Bio-HF risk score is a more comprehensive tool in that it incorporates the combinations of three biomarkers (NT-proBNP, hs-cTnT, and ST2) into the model. Nevertheless, our model, by only incorporating NT-proBNP, performed equally well in HFmrEF and HFpEF, and even better in HFrEF. Lastly, our comparisons to the MAGGIC, GWTG-HF, and BCN Bio-HF risk scores are pragmatic but potentially unfair since the predictors in our model were derived from the data we used for comparison. However, this bias should be largely lessened since the AUCs of our model were adjusted using the IECV technique.

Many of the prognostic factors identified in this study were already well established in previous studies. BUN and serum sodium were previously shown to have the highest predictive values among the most frequently used predictors and were also strongly associated with mortality in our study [6]. Most of the predictors in MAGGIC, such as age, SBP, COPD, diabetes, and serum creatinine, were further confirmed in our study. Like BIOSTAT-CHF [17], lower hemoglobin was associated with an increased risk of mortality. Consistent with several studies [29, 30], NT-proBNP was confirmed to be strongly associated with mortality. Inclusion of NT-proBNP is particularly an advantage of our model over the MAGGIC risk score. While it is still under debate whether the prognostic impact of NT-proBNP differs among HF subtypes [31], our study did not find the interaction between NT-proBNP and HF subtypes.

A novelty factor of this study is that we used a stratified Cox model to account for the cross-phenotype heterogeneity and this phenotype-specific model allowed both the baseline mortality risk as well as the effect of the prognostic variables to be different for each phenotype. Particularly, having a history of MI indicated increased mortality risk in HFrEF, while the effect of this variable was neutral in HFmrEF and HFpEF. It has been reported that ischemic etiology is associated with an increased risk of mortality in HFrEF but neutral in HFpEF [7, 32–35], and thus, it is not surprising that history of MI is more strongly associated with mortality in HFrEF. Consistent with Go et al. [12], history of diabetes was associated with higher mortality in HFrEF, but neutral in HFmrEF and HFpEF in our study. However, this was discordant with two previous studies [34, 36], in which diabetes was also associated with poor outcome in HFpEF. Consistent with MAGGIC, increased baseline SBP was associated with lower mortality in HFrEF, and this association disappeared in HFmrEF and HFpEF. Elevated serum creatinine was associated with lower mortality in HFmrEF, but neutral in HFrEF and HFpEF. This finding may suggest HFmrEF patients had a good diuretic response, which commonly showed an increase in serum creatinine, but still had good clinical outcomes [37]. Overall, we found the effect of the predictor variables to be similar for HFmrEF and HFpEF and more likely to be different for HFrEF, suggesting that HFmrEF is closer to HFpEF than to HFrEF.

The results of the IECV showed that our model discriminated well across the four different cohorts. Particularly in HFrEF, our model discriminated not only well in three cohorts close to routine clinical practice (BIOSTAT-index, BIOSTAT-validation, and TRIUMPH; AUC 0.76, 0.84, and 0.80), but equivalently well in the population from a RCT (COACH; AUC 0.76). In HFmrEF, the results suggested the Scottish patients in BIOSTAT-validation might have a different predictor-outcome association from other patients. In HFpEF, our model discriminated very well in BIOSTAT-validation and COACH, though not so well in BIOSTAT-index and TRIUMH. For BIOSTAT-index, this finding may be explained by the fact that HFpEF patients in this cohort were confined to NT-proBNP levels > 2000 pg/mL, resulting in a different population of HFpEF patients compared to the other three cohorts.

While differences in the baseline mortality risks among the four cohorts did not have a profound impact on model discrimination, model calibration was more heavily affected by this. For example, the predicted 1-year mortality risk was higher than the observed 1-year mortality risk for patients with HFrEF in BIOSTAT-index (Fig. 2), which is consistent with the lower observed mortality rate in this cohort relative to the other three cohorts (Table 2). Such discrepancies between the observed and predicted 1-year mortality risks attenuated after recalibration to the cohort-specific baseline risks, suggesting that more accurate predictions can be obtained by tailoring the parameter values of the baseline hazard functions to the baseline risk in the patient population to which the model is applied [28] (e.g., by taking the baseline hazard functions from the study cohort which has the closest observed outcome incidences).

The implication of our model relates to its ability to support bedside decision-making by complementing physician’s clinical judgment. Currently, treatment decisions in HF are based on population-averaged effects observed in RCTs. However, patients enrolled in RCTs can differ substantially in their risks of outcome and treatment effects are not necessarily homogeneous across the risk spectrum [38]. For example, in the PROTECT trial, the experimental treatment rolofylline was found to have a neutral effect in the treatment of acute HF with renal dysfunction [39]. However, in a subsequent post hoc analysis, Demissei et al. [4] found this treatment effect to be moderated by the predicted 180-day all-cause mortality risk, with rolofylline being beneficial in higher-risk patients but harmful in low-risk patients. These results suggest that there may still be a window of opportunity for rolofylline and other novel acute HF therapies that showed disappointing population-averaged effects, such as serelaxin [40], provided that a more targeted approach is implemented for the administering of these treatments. Risk prediction models, such as the one developed in this paper, are fundamental in moving forward such a more personalized approach in the treatment of acute HF.

Our study has several limitations. Firstly, the IPD meta-analysis included relatively small numbers of HFmrEF and HFpEF patients, which may hinder the generalizability of the results to other HFmrEF and HFpEF populations. Secondly, only variables that were measured in all four cohorts were considered as candidate predictors. Some of the more recently established prognostic markers such as ST2 [41] and Galectin-3 [42] could therefore not be included in our prognostic model. Finally, all the predictors including the ejection fraction were treated as time-fixed covariates, meaning that their values were assumed to remain constant across the prediction period. This is a limitation when large fluctuations in the values of the predictor variables are expected. However, given the relatively short prediction window and good model performance, it seems reasonable to treat the predictors as time-fixed for the present study.

## Conclusion

To conclude, using IPD meta-analysis, we were able to develop an HF phenotype stratified model for predicting 1-year mortality in patients hospitalized with acute HF that was generalizable across a range of European HF populations. Our model can therefore become a helpful tool in quantifying and classifying the prognosis of patients hospitalized with acute HF, allowing targeted treatment and management of those patients.

## Supplementary Information

**Additional file 1: Table S1.** Inclusion and exclusion criteria. **Table S2.** Extent of missing data in baseline characteristics. **Figure S1.** IECV-based calibration plots of the average predicted vs. observed 1-year mortality for the HFrEF patients in each cohort. **Figure S2.** IECV-based calibration plots of the average predicted vs. observed 1-year mortality for the HFmrEF patients in each cohort. **Figure S3.** IECV-based calibration plots of the average predicted vs. observed 1-year mortality for the HFpEF patients in each cohort. **Figure S4.** IECV-based calibration plots of the average predicted vs. observed 1-year mortality for the HFrEF patients in each cohort (with recalibration of the baseline mortality). **Figure S5.** IECV-based calibration plots of the average predicted vs. observed 1-year mortality for the HFmrEF patients in each cohort (with recalibration of the baseline mortality). **Figure S6.** IECV-based calibration plots of the average predicted vs. observed 1-year mortality for the HFpEF patients in each cohort (with recalibration of baseline mortality).

**Additional file 2.** Details of statistical modelling.

**Additional file 3.** Mathematical formulas for the prediction model and a patient example for the illustration of the calculation.

## Data Availability

The datasets analyzed during the current study are not publicly available, but are available from the corresponding author on reasonable request.

## Abbreviations

AUC: Area under the cumulative/dynamic time-dependent ROC
BUN: Blood urea nitrogen
CABG: Coronary artery bypass grafting
HF: Heart failure
HFmrEF: Heart failure with midrange ejection fraction
HFpEF: Heart failure with preserved ejection fraction
HFrEF: Heart failure with reduced ejection fraction
ICD: Implantable cardioverter defibrillator
IECV: Internal-external cross-validation
IPD: Individual participant data
MI: Myocardial infarction
NT-proBNP: N-terminal pro-B-type natriuretic peptide
PI: Prognostic index
RCT: Randomized controlled trial
SBP: Systolic blood pressure
SHFM: Seattle Heart Failure Model

## References

[1] Lesyuk W, Kriza C, Kolominsky-Rabas P. Cost-of-illness studies in heart failure: a systematic review 2004–2016. BMC Cardiovasc Disord. 2018;18. 10.1186/s12872-018-0815-3. 10.1186/s12872-018-0815-3 PMC5930493 29716540

[2] Heart-Failure-Summary-Report-2016-17.pdf. https://www.nicor.org.uk/wp-content/uploads/2018/11/Heart-Failure-Summary-Report-2016-17.pdf. Accessed 12 Sept 2019.

[3] Ziaeian B, Fonarow GC (2016). Epidemiology and aetiology of heart failure. Nat Rev Cardiol.

[4] Demissei BG, Postmus D, Liu LCY, Cleland JG, O’Connor CM, Metra M (2016). Risk-based evaluation of efficacy of rolofylline in patients hospitalized with acute heart failure — post-hoc analysis of the PROTECT trial. Int J Cardiol.

[5] Cao Q, Buskens E, Hillege HL, Jaarsma T, Postma M, Postmus D (2019). Stratified treatment recommendation or one-size-fits-all? A health economic insight based on graphical exploration. Eur J Health Econ.

[6] Ouwerkerk W, Voors AA, Zwinderman AH (2014). Factors influencing the predictive power of models for predicting mortality and/or heart failure hospitalization in patients with heart failure. J Am Coll Cardiol HF.

[7] Levy WC, Mozaffarian D, Linker DT, Sutradhar SC, Anker SD, Cropp AB (2006). The Seattle Heart Failure Model: prediction of survival in heart failure. Circulation..

[8] Pocock SJ, Ariti CA, McMurray JJV, Maggioni A, Køber L, Squire IB (2013). Predicting survival in heart failure: a risk score based on 39 372 patients from 30 studies. Eur Heart J.

[9] Meta-analysis Global Group in Chronic Heart Failure (MAGGIC) (2012). The survival of patients with heart failure with preserved or reduced left ventricular ejection fraction: an individual patient data meta-analysis. Eur Heart J.

[10] Lam CSP, Gamble GD, Ling LH, Sim D, Leong KTG, Yeo PSD (2018). Mortality associated with heart failure with preserved vs. reduced ejection fraction in a prospective international multi-ethnic cohort study. Eur Heart J.

[11] Jones NR, Roalfe AK, Adoki I, Hobbs FDR, Taylor CJ (2019). Survival of patients with chronic heart failure in the community: a systematic review and meta-analysis. Eur J Heart Fail.

[12] Go YY, Allen JC, Chia SY, Sim LL, Jaufeerally FR, Yap J (2014). Predictors of mortality in acute heart failure: interaction between diabetes and impaired left ventricular ejection fraction. Eur J Heart Fail.

[13] Voors AA, Anker SD, Cleland JG, Dickstein K, Filippatos G, van der Harst P (2016). A systems BIOlogy Study to TAilored Treatment in Chronic Heart Failure: rationale, design, and baseline characteristics of BIOSTAT-CHF: BIOSTAT-CHF: rationale and design. Eur J Heart Fail.

[14] NTR. https://www.trialregister.nl/trial/1783. Accessed 11 Apr 2019.

[15] Jaarsma T, van der Wal MH, Lesman-Leegte I, Luttik ML, Hogenhuis J, Veeger NJ, et al. Effect of moderate or intensive disease management program on outcome in patients with heart failure: Coordinating Study Evaluating Outcomes of Advising and Counseling in Heart Failure (COACH). Arch Intern Med. 2008;168:316–24. 10.1001/archinternmed.2007.83 18268174

[16] Ponikowski P, Voors AA, Anker SD, Bueno H, Cleland JGF, Coats AJS (2016). 2016 ESC Guidelines for the diagnosis and treatment of acute and chronic heart failure: the Task Force for the diagnosis and treatment of acute and chronic heart failure of the European Society of Cardiology (ESC) Developed with the special contribution of the Heart Failure Association (HFA) of the ESC. Eur Heart J.

[17] Voors AA, Ouwerkerk W, Zannad F, van Veldhuisen DJ, Samani NJ, Ponikowski P (2017). Development and validation of multivariable models to predict mortality and hospitalization in patients with heart failure: mortality and hospitalization models in heart failure. Eur J Heart Fail.

[18] Royston P, Parmar MKB, Sylvester R (2004). Construction and validation of a prognostic model across several studies, with an application in superficial bladder cancer. Statist Med.

[19] Therneau TM, Grambsch PM (2000). Modeling survival data: extending the Cox model.

[20] Wood AM, White IR, Royston P (2008). How should variable selection be performed with multiply imputed data?. Statist Med..

[21] Royston P, Altman DG (1994). Regression using fractional polynomials of continuous covariates: parsimonious parametric modelling. Appl Stat.

[22] Rizopoulos D, Molenberghs G, Lesaffre EMEH (2017). Dynamic predictions with time-dependent covariates in survival analysis using joint modeling and landmarking. Biom J.

[23] Blanche P, Dartigues J-F, Jacqmin-Gadda H (2013). Estimating and comparing time-dependent areas under receiver operating characteristic curves for censored event times with competing risks. Stat Med.

[24] Steyerberg EW, Harrell FE (2016). Prediction models need appropriate internal, internal–external, and external validation. J Clin Epidemiol.

[25] Peterson PN, Rumsfeld JS, Liang L, Albert NM, Hernandez AF, Peterson ED (2010). A validated risk score for in-hospital mortality in patients with heart failure from the American Heart Association Get With the Guidelines Program. Circ Cardiovasc Qual Outcomes.

[26] Lupón J, de Antonio M, Vila J, Peñafiel J, Galán A, Zamora E (2014). Development of a novel heart failure risk tool: the Barcelona Bio-Heart Failure Risk Calculator (BCN Bio-HF Calculator). PLoS One.

[27] Royston P, Altman DG (2013). External validation of a Cox prognostic model: principles and methods. BMC Med Res Methodol.

[28] Debray TPA, Moons KGM, Ahmed I, Koffijberg H, Riley RD (2013). A framework for developing, implementing, and evaluating clinical prediction models in an individual participant data meta-analysis. Statist Med..

[29] Pfister R, Diedrichs H, Schiedermair A, Rosenkranz S, Hellmich M, Erdmann E (2008). Prognostic impact of NT-proBNP and renal function in comparison to contemporary multi-marker risk scores in heart failure patients. Eur J Heart Fail.

[30] Wedel H, McMurray JJV, Lindberg M, Wikstrand J, Cleland JGF, Cornel JH (2009). Predictors of fatal and non-fatal outcomes in the Controlled Rosuvastatin Multinational Trial in Heart Failure (CORONA): incremental value of apolipoprotein A-1, high-sensitivity C-reactive peptide and N-terminal pro B-type natriuretic peptide. Eur J Heart Fail.

[31] Hamatani Y, Nagai T, Shiraishi Y, Kohsaka S, Nakai M, Nishimura K (2018). Long-term prognostic significance of plasma B-type natriuretic peptide level in patients with acute heart failure with reduced, mid-range, and preserved ejection fractions. Am J Cardiol.

[32] Tonje T, Claggett Brian L, Amil S, Susan C, Agarwal Sunil K, Wruck Lisa M (2017). Predicting risk in patients hospitalized for acute decompensated heart failure and preserved ejection fraction. Circ Heart Fail.

[33] Kasahara S, Sakata Y, Nochioka K, Tay WT, Claggett BL, Abe R (2019). The 3A3B score: the simple risk score for heart failure with preserved ejection fraction - a report from the CHART-2 Study. Int J Cardiol.

[34] Jones RC, Francis GS, Lauer MS (2004). Predictors of mortality in patients with heart failure and preserved systolic function in the Digitalis Investigation Group trial. J Am Coll Cardiol.

[35] Chen X, Savarese G, Dahlström U, Lund LH, Fu M. Age-dependent differences in clinical phenotype and prognosis in heart failure with mid-range ejection compared with heart failure with reduced or preserved ejection fraction. Clin Res Cardiol. 2019. 10.1007/s00392-019-01477-z. 10.1007/s00392-019-01477-z 30980205

[36] Komajda M, Carson PE, Hetzel S, McKelvie R, McMurray J, Ptaszynska A (2011). Factors associated with outcome in heart failure with preserved ejection fraction: findings from the Irbesartan in Heart Failure with Preserved Ejection Fraction Study (I-PRESERVE). Circ Heart Fail.

[37] Valente MAE, Voors AA, Damman K, Van Veldhuisen DJ, Massie BM, O’Connor CM (2014). Diuretic response in acute heart failure: clinical characteristics and prognostic significance. Eur Heart J.

[38] Kent DM, Paulus JK, van Klaveren D, D’Agostino R, Goodman S, Hayward R (2020). The Predictive Approaches to Treatment effect Heterogeneity (PATH) statement. Ann Intern Med.

[39] Massie BM, O’Connor CM, Metra M, Ponikowski P, Teerlink JR, Cotter G (2010). Rolofylline, an adenosine A1−receptor antagonist, in acute heart failure.

[40] Metra M, Teerlink JR, Cotter G, Davison BA, Felker GM, Filippatos G (2019). Effects of serelaxin in patients with acute heart failure. N Engl J Med.

[41] van Vark LC, Lesman-Leegte I, Baart SJ, Postmus D, Pinto YM, Orsel JG (2017). Prognostic value of serial ST2 measurements in patients with acute heart failure. J Am Coll Cardiol.

[42] van Vark LC, Lesman-Leegte I, Baart SJ, Postmus D, Pinto YM, de Boer RA, et al. Prognostic value of serial galectin-3 measurements in patients with acute heart failure. J Am Heart Assoc. 2017;6:e003700. 10.1161/JAHA.116.003700 PMC5778986 29187387

