# Supplementary material for: A heart failure phenotype stratified model for predicting 1-year mortality in patients admitted with acute heart failure: results from an individual participant data meta-analysis of four prospective European cohorts
Source: BMC Med. 2021 Jan 27;19:21. doi: 10.1186/s12916-020-01894-2 (PMC7839199; doi:10.1186/s12916-020-01894-2)
Supplement: Supplementary file 1 — Additional file 1: Table S1. Inclusion and exclusion criteria. Table S2. Extent of missing data in baseline characteristics. Figure S1. IECV-based calibration plots of the average predicted vs. observed 1-year mortality for the HFrEF patients in each cohort. Figure S2. IECV-based calibration plots of the average predicted vs. observed 1-year mortality for the HFmrEF patients in each cohort. Figure S3. IECV-based calibration plots of the average predicted vs. observed 1-year mortality for the HFpEF patients in each cohort. Figure S4. IECV-based calibration plots of the average predicted vs. observed 1-year mortality for the HFrEF patients in each cohort (with recalibration of the baseline mortality). Figure S5. IECV-based calibration plots of the average predicted vs. observed 1-year mortality for the HFmrEF patients in each cohort (with recalibration of the baseline mortality). Figure S6. IECV-based calibration plots of the average predicted vs. observed 1-year mortality for the HFpEF patients in each cohort (with recalibration of baseline mortality). [file 12916_2020_1894_MOESM1_ESM.docx]

Table S1. Inclusion and exclusion criteria

| **Study** | **Inclusion criteria** | **Exclusion criteria** |
| --- | --- | --- |
| BIOSTAT-index | 1. Aged ≥ 18 2. Cardiac dysfunction (LVEF≤40 or BNP>400 and/or NT-proBNP>2000) 3. Treated with oral or intravenous furosemide ≥40mg/day 4. Not previously treated with ACE inhibitors or ARBS and beta-blockers or receiving 50% of these drugs 5. Inpatient or outpatient | 1. Known diagnosis of septicaemia, acute myocarditis or hypertrophic obstructive, restrictive, or constrictive cardiomyopathy, heart transplant recipient or admitted for cardiac transplantation or left ventricular assist device surgery, anticipated need for surgery or any cardiovascular intervention within 4 weeks |
| BIOSTAT-validation | 1. Aged ≥ 18 2. Diagnosed with heart failure 3. A previous documented admission with heart failure requiring diuretic treatment 4. Be treated with furosemide≥20mg/day or equivalent 5. Not previously treated with ACE inhibitors or ARBS and beta-blockers or receiving 50% of these drugs 6. Anticipated initiation or uptitration of ACE/ARBs 7. Inpatient or outpatient |  |
| TRIUMPH | 1. Aged ≥ 18 2. Diagnosed with acute heart failure 3. Increased BNP or NT-proBNP 4. Treated with intravenous diuretics during the hospitalization 5. Evidence of sustained systolic or diastolic left ventricular dysfunction | 1. Heart failure precipitated by a non-cardiac condition 2. heart failure caused by severe valvular dysfunction or severe cardiac arrhythmias 3. Acute heart failure caused by an acute ST-segment elevation myocardial infarction or acute coronary syndrome 4. A planned coronary intervention 5. Patients with end-stage heart failure who are on the waiting list for cardiac transplantation 6. End-stage chronic kidney disease requiring dialysis 7. Non-cardiac condition associated with a life-expectancy <1 year |
| COACH | 1. Aged >18 2. Hospital admission for symptomatic chronic heart failure 3. Evidence for structural underlying heart disease | 1. Have undergone cardiac invasive intervention the last 6 months or planned the following 3 months 2. Are evaluated for heart transplantation |

Table S2. Extent of missing data in baseline characteristics

|  | BIOSTAT-index (n=1469) | BIOSTAT-validation (n=809) | TRIUMPH (n=372) | COACH (n=927) | Overall (n=3577) |
| --- | --- | --- | --- | --- | --- |
| **Characteristics** |  |  |  |  |  |
| Sex | 0 | 0 | 0 | 0 | 0 |
| Age | 0 | 0 | 0 | 0 | 0 |
| BMI | 21 (1.4) | 15 (1.9) | 24 (6.5) | 7 (0.8) | 67 (1.9) |
| Systolic blood pressure | 4 (0.3) | 5 (0.6) | 3 (0.8) | 5 (0.5) | 17 (0.5) |
| Diastolic blood pressure | 4 (0.3) | 5 (0.6) | 3 (0.8) | 5 (0.5) | 17 (0.5) |
| Heart rate | 4 (0.3) | 10 (1.2) | 3 (0.8) | 6 (0.6) | 23 (0.6) |
| Previous HF hospitalization | 0 | 15 (1.9) | 0 | 0 | 15 (0.4) |
| NYHA class | 40 (2.7) | 0 | 19 (5.1) | 8 (0.9) | 67 (1.9) |
| **Medical history** |  |  |  |  |  |
| Myocardial infarction | 0 | 1 (0.1) | 0 | 0 | 1 (0) |
| CABG | 0 | 1 (0.1) | 0 | 0 | 1 (0) |
| Atrial fibrillation | 0 | 5 (0.6) | 0 | 0 | 5 (0.1) |
| ICD/Pacemaker | 0 | 0 | 2 (0.5) | 0 | 2 (0.1) |
| COPD | 0 | 6 (0.7) | 1 (0.3) | 0 | 7 (0.2) |
| Peripheral arterial disease | 0 | 25 (3.1) | 1 (0.3) | 0 | 26 (0.7) |
| Stroke | 0 | 7 (0.9) | 0 | 0 | 7 (0.2) |
| Diabetes | 0 | 5 (0.6) | 1 (0.3) | 0 | 6 (0.2) |
| **Medication** |  |  |  |  |  |
| β-blocker use | 0 | 0 | 18 (4.8) | 0 | 18 (0.5) |
| ACE/ARBs use | 0 | 0 | 18 (4.8) | 0 | 18 (0.5) |
| Diuretics use | 0 | 0 | 18 (4.8) | 0 | 18 (0.5) |
| **Labratory** |  |  |  |  |  |
| Haemoglobin | 23 (1.6) | 5 (0.6) | 5 (1.3) | 13 (1.4) | 46 (1.3) |
| Hematocrit | 52 (3.5) | 4 (0.5) | 19 (5.1) | 156 (16.8) | 231 (6.5) |
| Serum potassium | 21 (1.4) | 5 (0.6) | 1 (0.3) | 1 (0.1) | 28 (0.8) |
| Serum sodium | 18 (1.2) | 4 (0.5) | 2 (0.5) | 1 (0.1) | 25 (0.7) |
| Serum creatinine | 11 (0.7) | 3 (0.4) | 2 (0.5) | 0 | 16 (0.4) |
| BUN | 161 (11) | 3 (0.4) | 3 (0.8) | 72 (7.8) | 239 (6.7) |
| NT-proBNP | 743 (50.6) | 20 (2.5) | 24 (6.5) | 401 (43.3) | 1188 (33.2) |


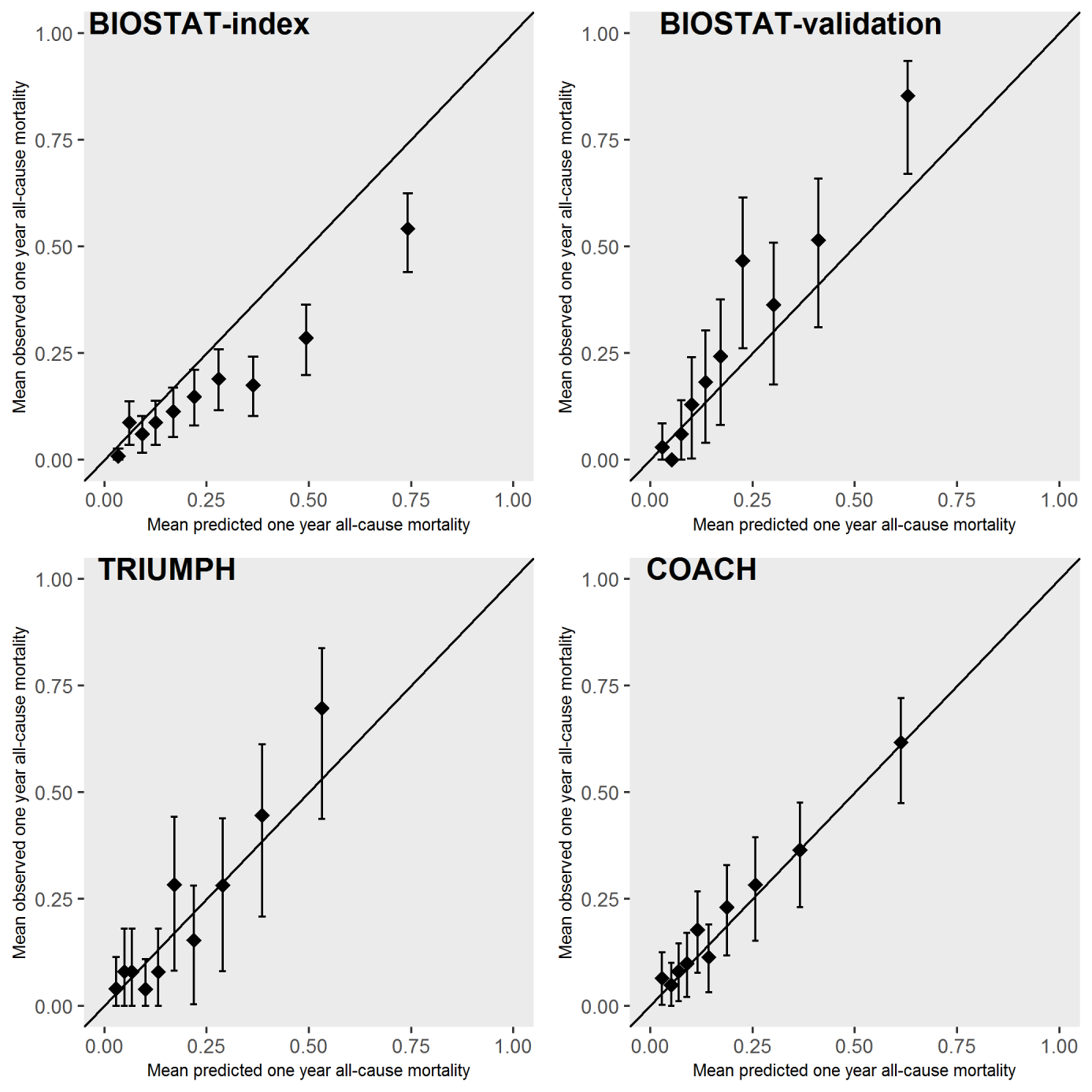


Figure S1. IECV-based calibration plots of the average predicted vs. observed 1-year mortality (Kaplan-Meier estimate + corresponding 95% confidence interval) by deciles of predicted 1-year mortality for the HFrEF patients in each cohort (without recalibration)


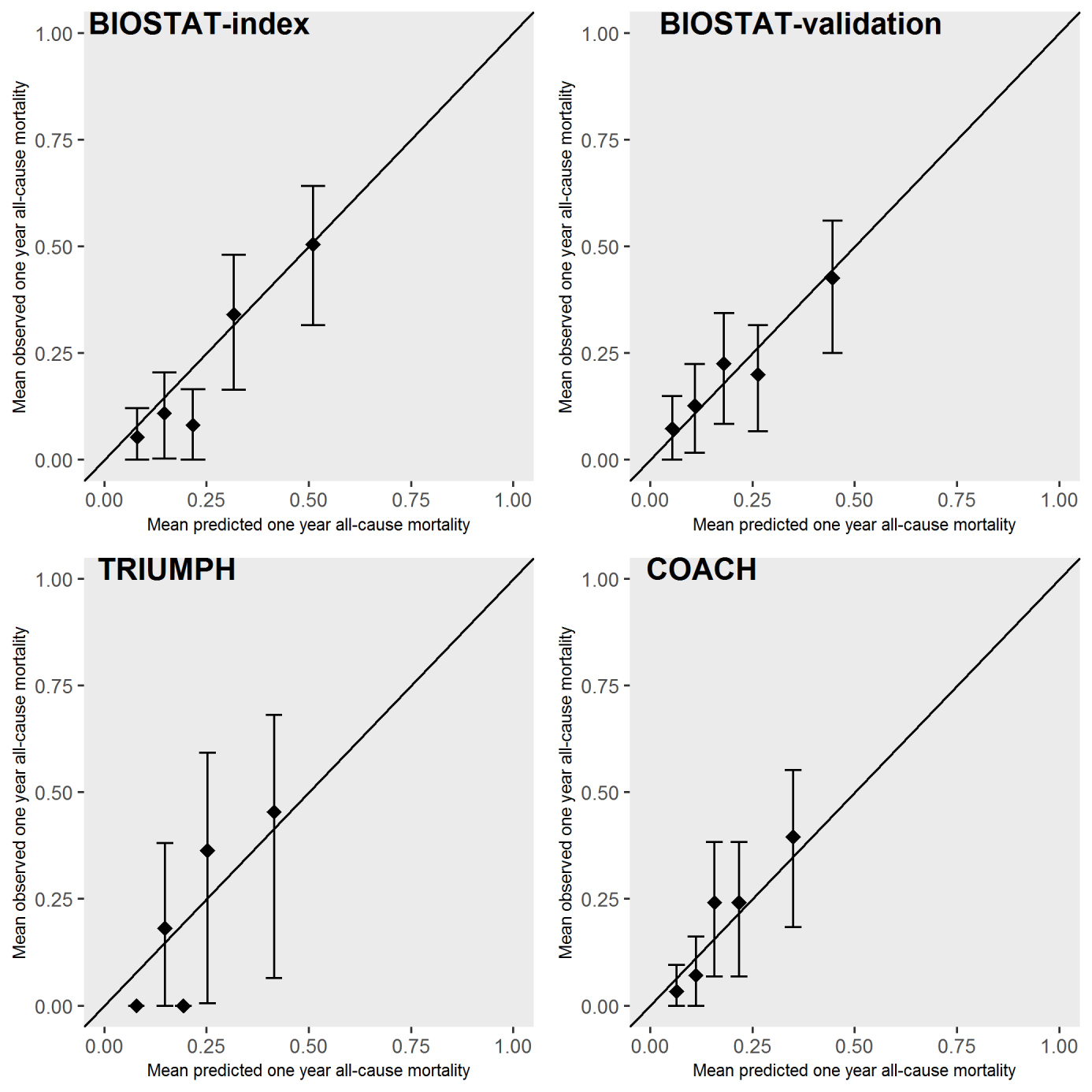


Figure S2. IECV-based calibration plots of the average predicted vs. observed 1-year mortality (Kaplan-Meier estimate + corresponding 95% confidence interval) by quintiles of predicted 1-year mortality for the HFmrEF patients in each cohort (without recalibration)


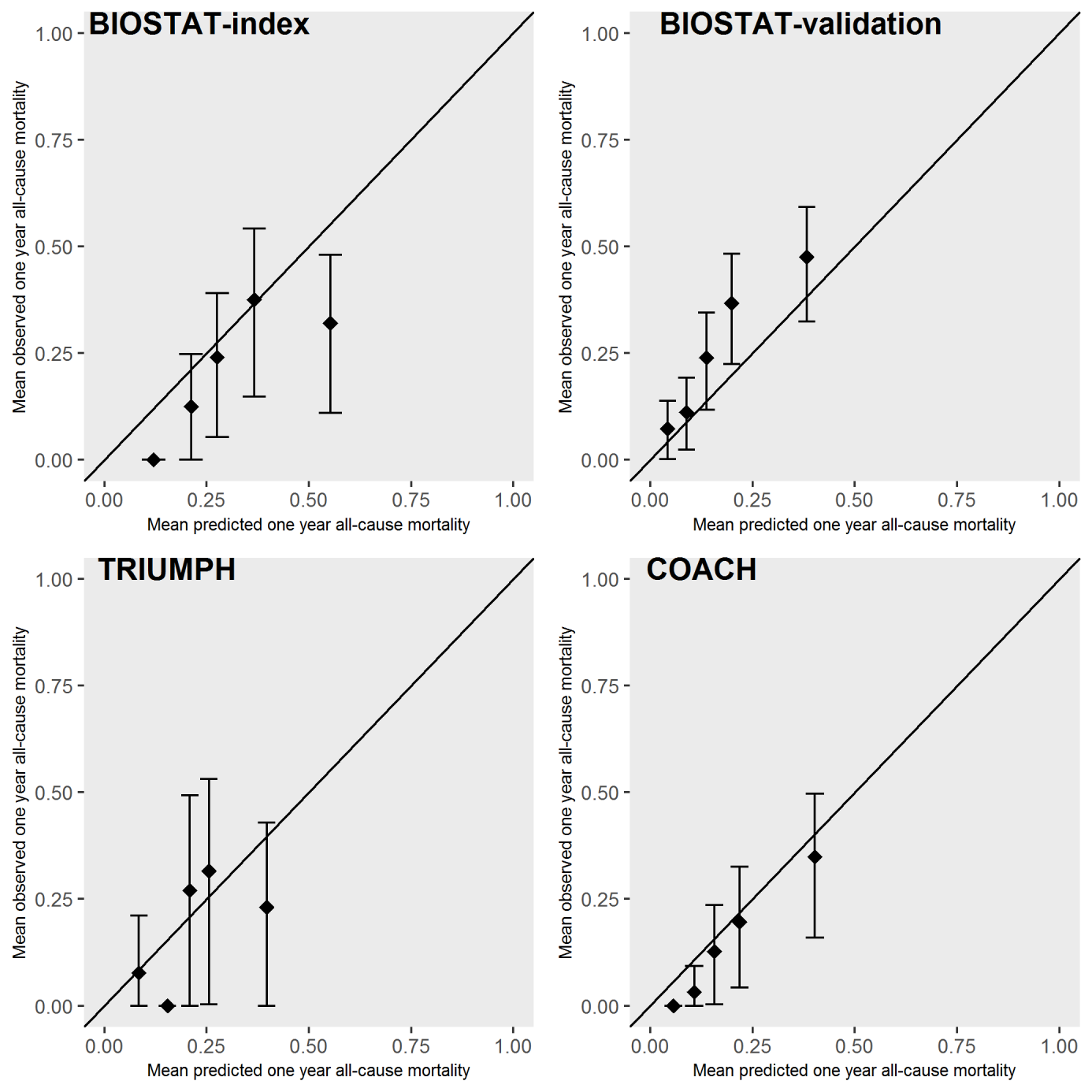


Figure S3. IECV-based calibration plots of the average predicted vs. observed 1-year mortality (Kaplan-Meier estimate + corresponding 95% confidence interval) by quintiles of predicted 1-year mortality for the HFpEF patients in each cohort (without recalibration)


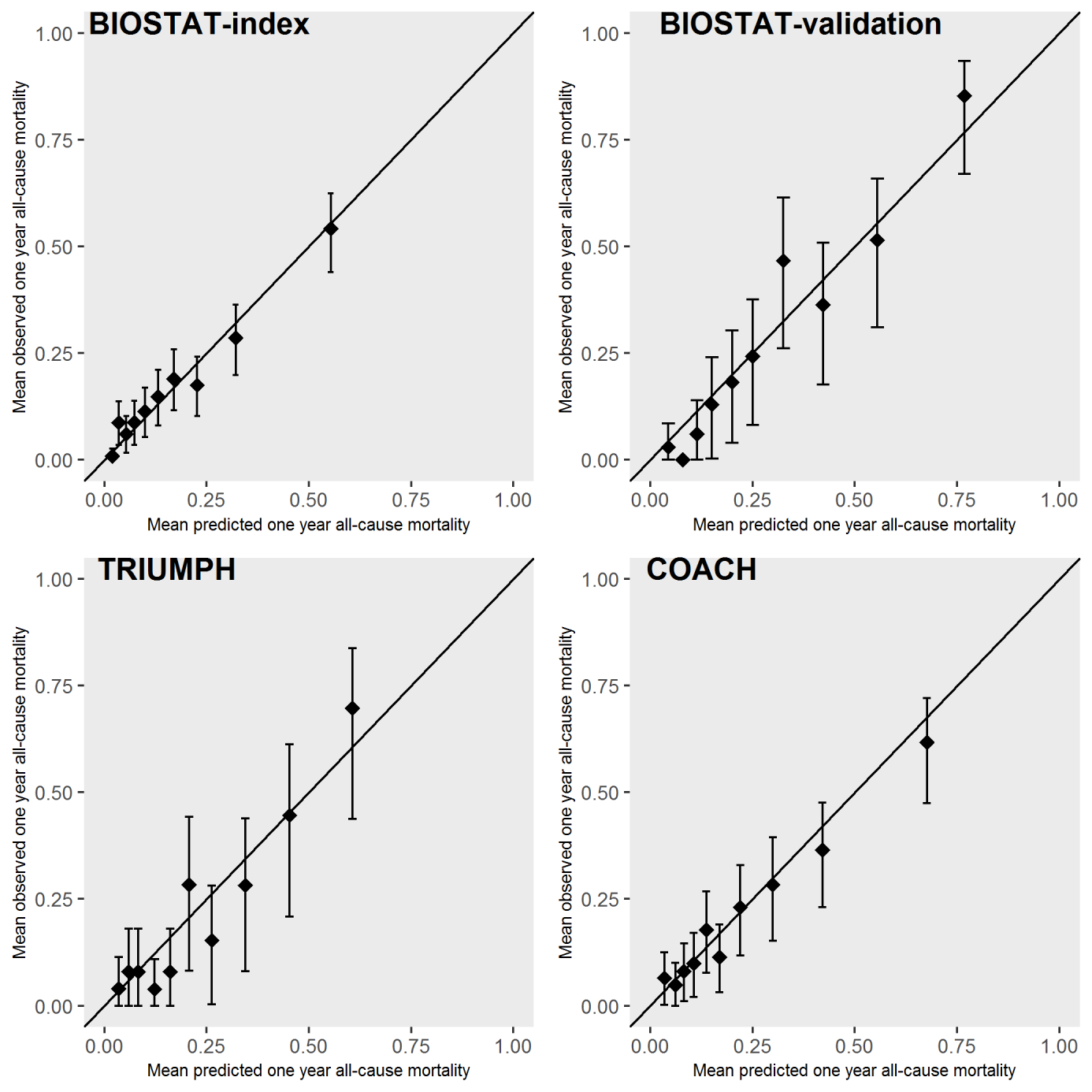


Figure S4. IECV-based calibration plots of the average predicted vs. observed 1-year mortality (Kaplan-Meier estimate + corresponding 95% confidence interval) by deciles of predicted 1-year mortality for the HFrEF patients in each cohort (with recalibration of the baseline mortality)


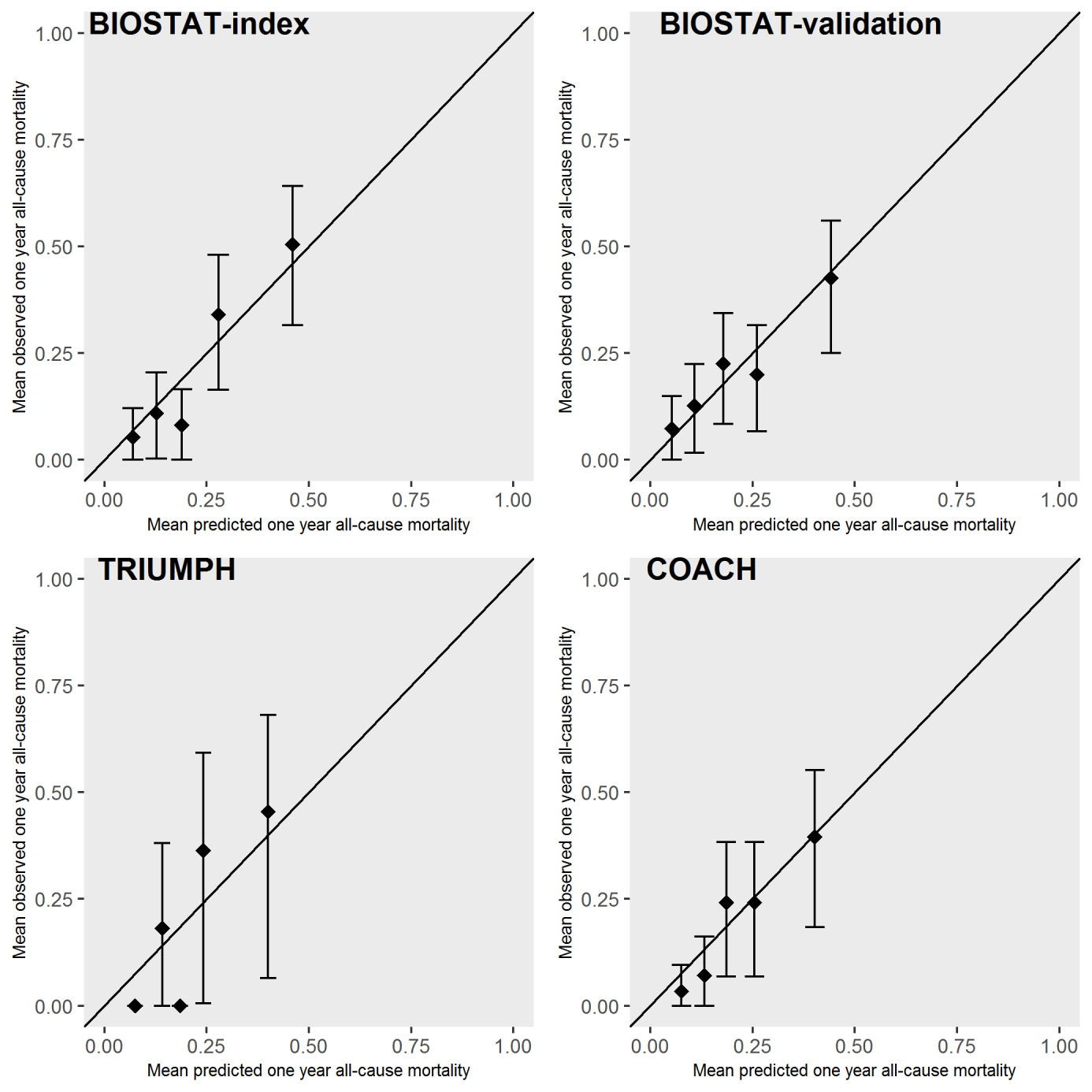


Figure S5. IECV-based calibration plots of the average predicted vs. observed 1-year mortality (Kaplan-Meier estimate + corresponding 95% confidence interval) by quintiles of predicted 1-year mortality for the HFmrEF patients in each cohort (with recalibration of the baseline mortality)


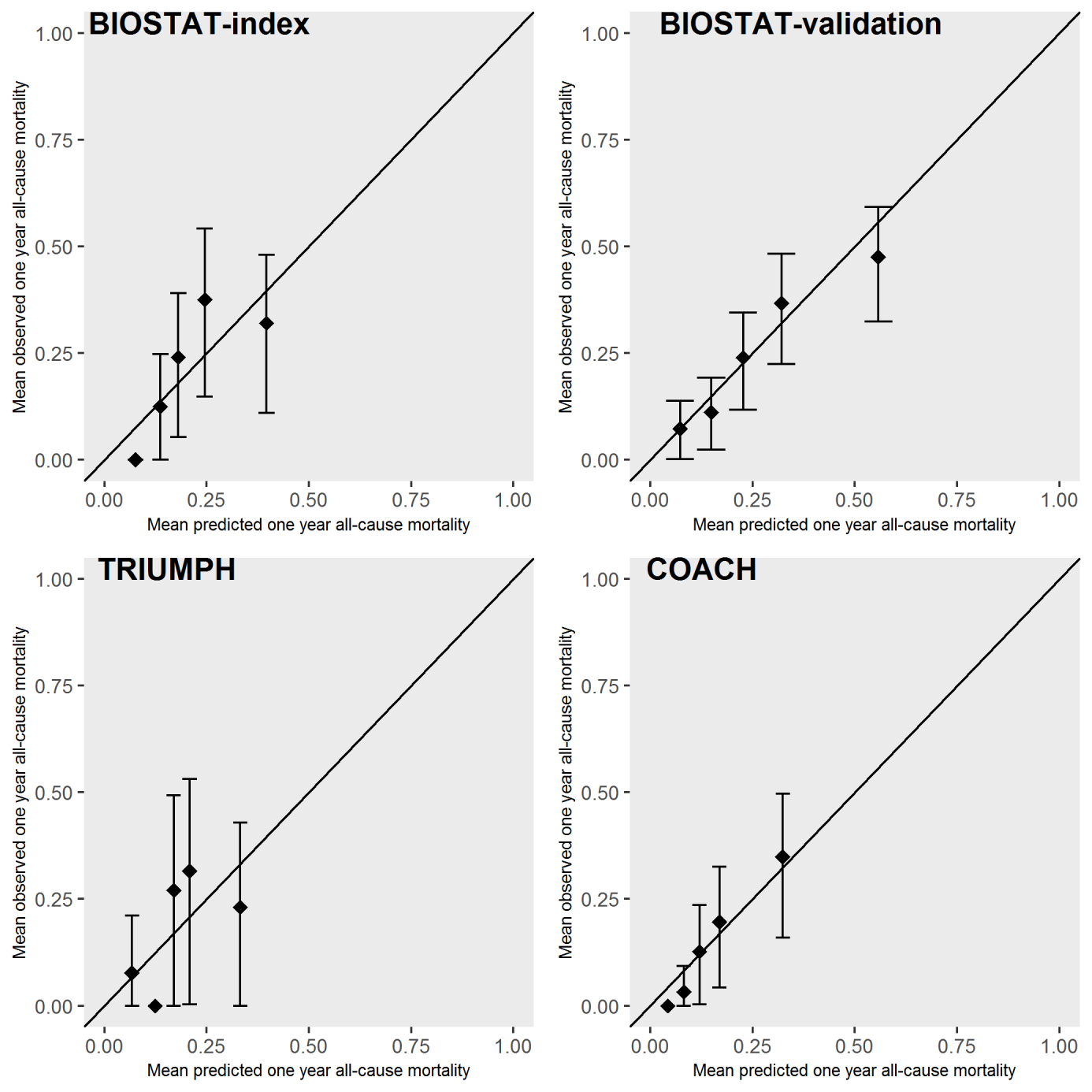


Figure S6. IECV-based calibration plots of the average predicted vs. observed 1-year mortality (Kaplan-Meier estimate + corresponding 95% confidence interval) by quintiles of predicted 1-year mortality for the HFpEF patients in each cohort (with recalibration of baseline mortality)
