# Supplementary material for: A heart failure phenotype stratified model for predicting 1-year mortality in patients admitted with acute heart failure: results from an individual participant data meta-analysis of four prospective European cohorts
Source: BMC Med. 2021 Jan 27;19:21. doi: 10.1186/s12916-020-01894-2 (PMC7839199; doi:10.1186/s12916-020-01894-2)
Supplement: Supplementary file 2 — Additional file 2. Details of statistical modelling. [file 12916_2020_1894_MOESM2_ESM.docx]

**Details of statistical modeling**

Our prognostic model consists of two parts: a prognostic index derived by a Cox model and heart failure (HF) subtype (HFrEF, HFmrEF, and HFpEF) specific baseline hazards derived by a Weibull model.

Step 1:

A Cox proportional hazards regression stratified by study *u* and HF subtype *v* was used to estimate the effects of the predictor variables, with the following hazard function:

$h_{uv}\left( t \right)=h_{0uv}(t)exp(\sum_{j=1}^{m} \beta_{j}x_{ij})$,

Where $x_{ij}$is the value of patients *i* on predictor *j* and $\beta_{j}$ the respective regression coefficient and $h_{0uv}(t)$ the unspecified baseline hazard for study *u* and HF subtype *v*.

Step 2:

A Weibull proportional hazards regression stratified by HF subtype *v* was used to estimate baseline hazards, with the following hazard function:

$\tilde{h}_{v}\left( t \right)=\tilde{h}_{0v}(t)exp(\sum_{j=1}^{m} \beta_{j}x_{ij})$,

with the baseline hazard function $\breve{h}_{0v}\left( t \right)$ defined as

$\tilde{h}_{ov}\left( t \right)={(\frac{1}{b_{v}})}^{\rho_{v}}\rho_{v}t^{\rho_{v}-1}$.

Where $\sum_{j=1}^{m} \beta_{j}x_{ij}$ is already estimated from the previous Cox model in step 1 and $b_{v}$ is the baseline scale parameter for HF subtype *v*. The shape parameter $\rho_{v}$ was assumed to remain constant across patients with the same HF subtype.

Finally, survival can be calculated as follows:

$\tilde{S}_{v}\left( t \right)=\exp\left( -\int_{0}^{t} \tilde{h}_{v}(t) \right)$.
