# Supplementary material for: A heart failure phenotype stratified model for predicting 1-year mortality in patients admitted with acute heart failure: results from an individual participant data meta-analysis of four prospective European cohorts
Source: BMC Med. 2021 Jan 27;19:21. doi: 10.1186/s12916-020-01894-2 (PMC7839199; doi:10.1186/s12916-020-01894-2)
Supplement: Supplementary file 3 — Additional file 3. Mathematical formulas for the prediction model and a patient example for the illustration of the calculation. [file 12916_2020_1894_MOESM3_ESM.docx]

**Prognostic index (PI)**

The PI for a specific patient is calculated as the linear combination of the regression coefficients (Table 2) and values of the corresponding (transformed) predictors for that patient as the following formula:

$PI=0.023*Age+0.298*COPD+0.360*NYHA1+0.298*NYHA2-0.164*Haemoglobin-0.032*Sodium+0.335*Serum urea nitrogen+0.294*NTproBNP-0.029*SBP+0.020*SBP*HFmrEF+0.023*SBP*HFpEF+0.037*Creatinine-0.405*Creatinine*HFmrEF-0.246*Creatinine*HFpEF+0.430*Myocardial infarction-0.462*Myocardial infarction*HFmrEF-0.646*Myocardial infarction*HFpEF+0.265*Diabetes-0.441*Diabetes*HFmrEF-0.342*Diabetes*HFpEF$, **(Formula 1)**

- Age (year)
- COPD: Yes = 1, No = 0
- Diabetes: Yes = 1, No = 0
- Myocardial infarction: Yes = 1, No = 0
- NYHA class: NYHA I/II = (NYHA1=NYHA2=0); NYHA III = (NYHA1=1 and NYHA2=0); NYHA IV = (NYHA1=0 and NYHA2=1)
- SBP (mmHg): if SBP > 130mmHg, truncated at 130
- Haemoglobin (mmol/L)
- Sodium (mmol/L)
- Serum urea nitrogen (mmol/L), NT-proBNP (ng/L), Creatinine (umol/L): transformed to (2-log)
- HF subtype: HFrEF, left ventricular ejection fraction (LVEF) < 40%; HFmrEF, LVEF 40-49%; HFpEF, LVEF ≥ 50%. Coding: HFrEF = (HFmrEF=HFpEF=0); HFmrEF = (HFmrEF=1 and HFpEF=0); HFpEF = (HFmrEF=0 and HFpEF=1)

**One Year Mortality Calculator**

The final mathematic formula for calculating 1-year mortality for patient $i$ is

$F_{i}\left( t \right)=1-S_{i}\left( t \right)=1-\left[ \exp[{-\left( \frac{t}{b} \right)}^{a}] \right]^{\exp\left( {PI}_{i} \right)},$ **(Formula 2)**

where shape parameter $a$ is 0.82, 0.74 and 0.73 for HFrEF, HFmrEF and HFpEF, and scale parameter $b$ is 299, 69 and 461 for HFrEF, HFmrEF and HFpEF, and $t=365$ since 1-year mortality is of our interest, ${PI}_{i}$ is the prognostic index of patient $i$ calculated using Formula 1.

Thus, full model for predicting 1-year mortality was as follows:

For HFrEF, $F_{i}\left( 365 \right)=1-0.308^exp({PI}_{i})$,

For HFmrEF, $F_{i}\left( 365 \right)=1-0.032^exp({PI}_{i})$,

For HFpEF, $F_{i}\left( 365 \right)=1-0.430^exp({PI}_{i})$.

**Patient Example for Model Use**

The following example explains how the model can be used in clinical practice: Suppose a 73 years old male patient was admitted into hospital because of acute heart failure. He had a myocardial infarction, but no diabetes or COPD medical history. Doctors classified him as a NYHA IV heart failure patient according to the severity of his symptoms. The left ventricular ejection fraction of this patient was 37%. His blood pressure was 125/85 mmHg. His blood tests show the following results:

- Haemoglobin (mmol/L): 8.8
- Sodium (mmol/L): 135
- Serum urea nitrogen (mmol/L): 7.9
- NT-proBNP (ng/L): 4787
- Creatinine (umol/L): 118

Because LVEF is smaller than 40%, this patient is defined as HFrEF. Based on the above information, the user can first use Formula 1 to calculate the PI of this patient as follows:

$PI=0.023*73+0.298*0+0.360*0+0.298*1-0.164*8.8-0.032*135+0.335*\log_{2} 7.9+0.294*\log_{2} 4787-0.029*125+0.020*125*0+0.023*125*0+0.037*\log_{2} 118-0.405*\log_{2} 118*0-0.246*\log_{2} 118*0+0.430*1-0.462*1*0-0.646*1*0+0.265*0-0.441*0*0-0.342*0*0=-2.133$

The user then uses Formula 2 to calculate the 1-year mortality by inputting the PI:

$$F_{i}\left( t \right)=1-S_{i}\left( t \right)=1-\left[ \exp\left[ {-\left( \frac{t}{b} \right)}^{a} \right] \right]^{\exp\left( {PI}_{i} \right)}=1-\left[ \exp\left[ -\left( \frac{365}{299} \right)^{0.82} \right] \right]^{\exp\left( -2.133 \right)}=1-{0.308}^{\exp\left( -2.133 \right)}=13.02\%$$

The relation between the PI and 1-year moryality is depicted in Figure 1. Given the prognostic index equalling to -2.133 (close to median value of the prognostic index in HFrEF), this patient can be classified as middle-risk in HFrEF. Furthermore, the user can also get a rough estimate of 1-year mortality risk by locating the prognostic index on the 1-year mortality axis (Figure 1). We are also thriving to develop a Shiny App for our prediction model to facilitate its clinical usage.
